# Differential nuclear localization of complexes may underlie *in vivo* intrabody efficacy in Huntington's disease

**DOI:** 10.1093/protein/gzu041

**Published:** 2014-10

**Authors:** D.C. Butler, A. Snyder-Keller, E. De Genst, A. Messer

**Affiliations:** 1 Wadsworth Center, New York State Department of Health, Department of Biomedical Sciences, University at Albany, Albany, NY 12208, USA; 2 Department of Chemistry, University of Cambridge, Lensfield Road, Cambridge CB2 1EW, UK; 3 Neural Stem Cell Institute, Rensselaer, NY 12144, USA

**Keywords:** Huntington's disease, intrabody, nuclear, polyglutamine, single-chain Fv (scFv)

## Abstract

Intrabodies offer attractive options for manipulating the protein misfolding that triggers neurodegenerative diseases. In Huntington's disease, where the expanded polyglutamine tract in the extreme N-terminal region of huntingtin exon1 misfolds, two lead intrabodies have been selected against an adjacent peptide, using slightly different approaches. Both are effective at preventing aggregation of a reporter fragment in transient co-transfection assays. However, after intracranial delivery to mutant mouse brains, VL12.3, which is mainly localized to the nucleus, appears to accelerate the mutant phenotype, while C4 scFv, which is largely cytoplasmic, shows partial phenotypic correction. This comparison highlights parameters that could inform intrabody therapeutics for multiple proteostatic diseases.

## Introduction

**Fig. 1.**
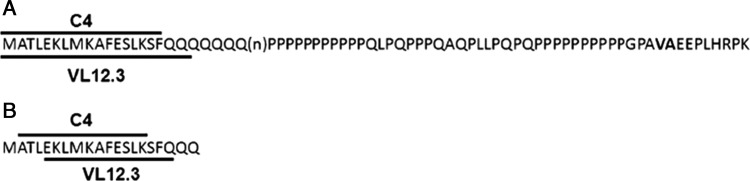
Diagram of Huntingtin (Htt) exon1 with the selection (A) and the binding sites (B) of C4 scFv and V_L_12.3.

Huntington's disease (HD) is caused by the misfolding of the N-terminal exon1 fragment of the protein product of a mutant gene encoding an expanded (>36 copies) CAG trinucleotide repeat (Hatters, 2008; Imarisio *et al.*, 2008). The resultant expanded polyglutamine (polyQ) has a high propensity to adopt β-sheet structure, causing the protein to aggregate into highly ordered amyloid fibrils and to interact abnormally with a variety of other proteins. Striatal neurons are particularly vulnerable to this cellular disruption, and show a phenotype that includes neuronal intranuclear inclusions and transcriptional dysregulation. Altering the protein context of the polyQ had the potential to alter the misfolding kinetics; therefore, an intrabody approach seemed justified. Using the Sheets single-chain Fv (scFv) library from the Marks lab (Sheets *et al.*, 1998), a selection against the N-terminal 17AA adjacent to the polyQ (Fig. 1A) was used to generate a candidate construct, C4 scFv, that has shown efficacy against the aggregation and/or toxicity phenotypes in cellular, organotypic slice culture, Drosophila and transgenic mouse models of HD (Lecerf *et al.*, 2001; Murphy and Messer, 2004; Miller *et al.*, 2005; Wolfgang *et al.*, 2005; Snyder-Keller *et al.*, 2010; Butler and Messer, 2011).

In an effort to further enhance efficacy, a subsequent selection vs AAs 1–20 (Fig. 1A) including the first 3 glutamine residues was performed by Colby *et al.*, using a yeast surface display library. After multiple rounds of engineering to improve intracellular stability and increase affinity, the resultant V_L_12.3 was able to correct misfolded Huntingtin exon1 (Httex1) protein at lower concentrations than scFv when tested in co-transfection assays (Colby *et al.*, 2004a,b). However, *in vivo* efficacy was sub-optimal, including accelerated onset of multiple phenotypic markers (Southwell *et al.*, 2008, 2009).

V_L_12.3, particularly when complexed with its Httex1 target, appears to reside primarily in the nucleus (Southwell *et al.*, 2008, 2009). Given the multiple observations of a potential mode of HD toxicity via nuclear processes, including a more aggressive disease phenotype when an Httex1 transgene was directly fused to a nuclear localization signal (NLS; Davies *et al.*, 1997; DiFiglia *et al.*, 1997; Steffan *et al.*, 2000; Benn *et al.*, 2005, 2008; Atwal *et al.*, 2007), we now show that C4 scFv is primarily cytoplasmic when compared with V_L_12.3 over a range of assay systems, and confirm C4 scFv cytoplasmic localization in brain cells by confocal microscopy. This intrabody also does not accelerate overall disease parameters of weight loss and early death. These data illustrate the critical merging of improved understanding of the disease mechanisms with cellular assays to design more powerful early-stage screening protocols.

## Materials and methods

### Expression plasmids

cDNAs encoding the intrabodies in pAAV—C4 scFv (GenBank accession number EU490426) and V_L_12.3—were used as previously described (Colby *et al.*, 2004a,b; Kvam *et al.*, 2009). For the nuclear export signal (NES) control experiments, DNA was polymerase chain reaction (PCR) amplified using complementary primers that encoded mitogen-activated protein kinase kinase NES. The resulting PCR product was ligated into pAAV-MCS (Stratagene) at the corresponding Xba1 and HindIII restriction sites using standard cloning techniques. The resulting expression plasmid cassette was the following: pAAV-MCS-Kozak sequence-C4 scFv-NES-HA-stop or pAAV-MCS-Kozak sequence-V_L_12.3NES-HA-stop. Human Httex1 with 72 polyglutamine repeat lengths was labeled with enhanced green fluorescent protein, Httex1-72Q-EGFP, and expressed with pcDNA3.1(-) plasmid vector. To label nuclei in live cells, a NLS-tagged monomeric red fluorescent protein (RFP-NLS) described by Kvam *et al.* (2009) was cotransfected in with Httex1-72Q-EGFP and intrabody. All expression plasmids were prepared using EndoFree Plasmid Maxi (Qiagen) and confirmed by DNA sequencing.

### Cell culture and transfection

Undifferentiated ST14A cells were cultured at the permissive temperature (33°C), according to standard protocols (Ehrlich *et al.*, 2001). ST14A cells were transiently transfected with jetPEI DNA transfection reagent (Polyplus Transfection Inc.) as previously described (Kvam *et al.*, 2009). For all transfections, intrabody plasmids were applied at equal (1 : 1) ratios to Httex1 plasmids, and cells were analyzed at either 24 or 48 h post-transfection.

### Live-cell imaging

ST14A cells transfected with Httex1-72Q-eGFP reporters were imaged directly in 6-well culture dishes using an Olympus IX70 inverted microscope equipped with an Olympus IXFLA Inverted Reflected Light Fluorescence Observation attachment and RGB Mirror Cube filter wheel (Olympus). Cells were observed without fixation using a 406 lens, and images were captured at either 24 or 48 h post-transfection with a SPOT RT Color CCD camera using SPOT Advanced software (Diagnostic Instruments). Digital images were overlayed using Adobe Photoshop.

### Nuclear vs cytoplasmic counting

To quantify the subcellular differences in the expression of Httex1-72Q-eGFP when co-transfected with the intrabodies, the extent to which the GFP signal was found in cytoplasm was counted in ST14A cells cotransfected with Httex1-72Q-EGFP, RFP-NLS and either C4 scFv or V_L_12.3. A two proportion *z*-test was then used to determine if a significant difference in the proportion of cells with cytoplasmic GFP existed between groups.

### *In vivo* weight and survival studies: bilateral AAV-C4 injections into R6/1 mice

AAV2/1 C4 scFv-HA was produced at the University of Iowa Vector core (Dr. B. Davidson, director), as previously described. The B6.HD6/1 mice, genotype verified by PCR of tail biopsy, were bilaterally intrastriatally injected at 7–9 weeks of age, with 2 μl of AAV2/1 anti-HTT C4 scFv (10e12 Vg/ml)/side. Injected mice were housed at no more than three per cage with littermates, observed daily and weighed weekly. All animal procedures were approved by the Wadsworth Center Institutional Animal Care and Use Committee. The comparisons to intrastriatal injections of V_L_12.3 were made to the published data by Southwell *et al.* (2009).

### Immunostaining and confocal imaging after intrastriatal injection

HDR6/1 mice were injected at 11 weeks of age. At 20 weeks of age, injected mice were perfused with 4% paraformaldehyde, brains stored in the same fixative for 20 h, then permeated in 15% sucrose prior to the preparation of 30-micron frozen sections on a sliding microtome. Sections were selected for fluorescence double-labeling using antibodies to mutant Huntingtin (mHtt) protein (EM48) and Alexa 488-labeled secondary antibodies, and then antibodies to HA and Alexa 594-labeled secondary antibodies, sequentially. Image capture and analysis were performed on a Leica TCS SP5 confocal microscope.

## Results and discussion

**Fig. 2.**
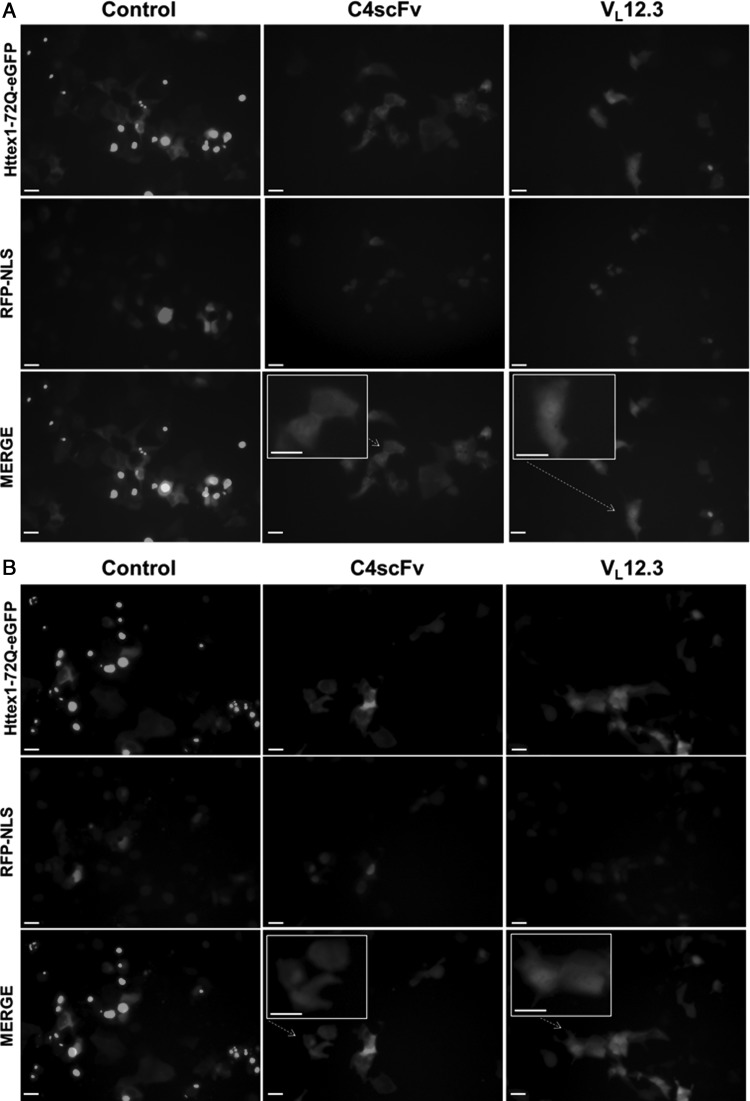
Httex1-72Q-eGFP is predominately cytoplasmic in cells cotransfected with either empty vector control or C4 scFv compared to V_L_12.3 at 24H (A) and 48H time points (B). ST14A cells were co-transfected with Httex1-72Q-eGFP and RFP-NLS (to label nuclei in live cells), and either empty vector control, C4 scFv or V_L_12.3. Live imaging was performed at 24 and 48 h. Bar = 20 μm. A color version of this figure is available as supplementary data at *PEDS* Online.

We have noted over several years of cellular and *in vivo* experiments that the localization of Httex1-72Q-eGFP differs between C4 scFv and V_L_12.3 transductions, with the former clearly much more cytoplasmic. In order to specifically examine the differences between the two intrabodies *in situ*, a set of live imaging experiments was performed. ST14A cells were transfected with Httex1-72Q-GFP and either C4 scFv, V_L_12.3, or empty vector control. To label nuclei in live cells, we additionally transfected a NLS-tagged monomeric RFP in these cotransfection experiments. While not all cells were transduced with all three plasmids, we have previously reported that multiple plasmids appear to transduce cells in common when delivered together (LeCerf *et al.*, 2001; Miller *et al.*, 2005). With live cell imaging at 24 and 48 h, the Httex1-72Q-eGFP combined with an empty vector control appears primarily aggregated with very few cells displaying diffuse cytoplasmic Httex1-72Q-GFP expression (Fig. 2A and B; color supplemental Fig. S2A and B). Cells co-transfected with C4 scFv displayed a diffuse Httex1-72Q-eGFP expression in the cytoplasm with lower levels in nuclei in 62.5% of the cells (Table I). Rare aggregates are only located in the cytoplasm (Supplementary Fig. S1B). In contrast, with V_L_12.3 co-transfection, only 10.5% of the Httex1-72Q-eGFP cotransfected cells displayed predominantly cytoplasmic GFP (Table I). The diffuse material is noticeably stronger in the nucleus of many of these cells (Fig. 2A and B, Supplementary Fig. S1C). The clarity of the nucleus in these observations is primarily determined by the plane of focus and/or how recently the cells have divided; therefore, we have grouped all of the non-cytoplasmic GFP cells together for the purposes of this analysis. This difference in subcellular localization is consistently observed over a range of experimental protocols. An example from one of a large series of separate experiments showing the same localization pattern is shown in Supplementary Fig. S1C. While the V_L_12.3 co-transfection is quite efficient at reducing the visible aggregation, any observable aggregates appeared to be nuclear (Supplementary Fig. S3C). As an additional control, we fused the MAPKK-NES signal to both C4 scFv and VL12.3. These fusions successfully removed Httex1-72Q-GFP from the nucleus in both cases, demonstrating that the intrabody differences are not secondary to changes in the nuclear membranes (Supplementary Figs S3B and D).

**Table I.** The proportion of cells that have well-defined cytoplasmic GFP is significantly higher in cells transfected with C4 scFv compared with V_L_12.3. (*P* < 0.001)

	Predominantly cytoplasmic GFP	Uniform or more nuclear GFP	Total	Proportion of cells with cytoplasmic GFP
C4 scFv	35	22	56	62.5%
V_L_12.3	6	51	57	10.5%

**Fig. 3.**
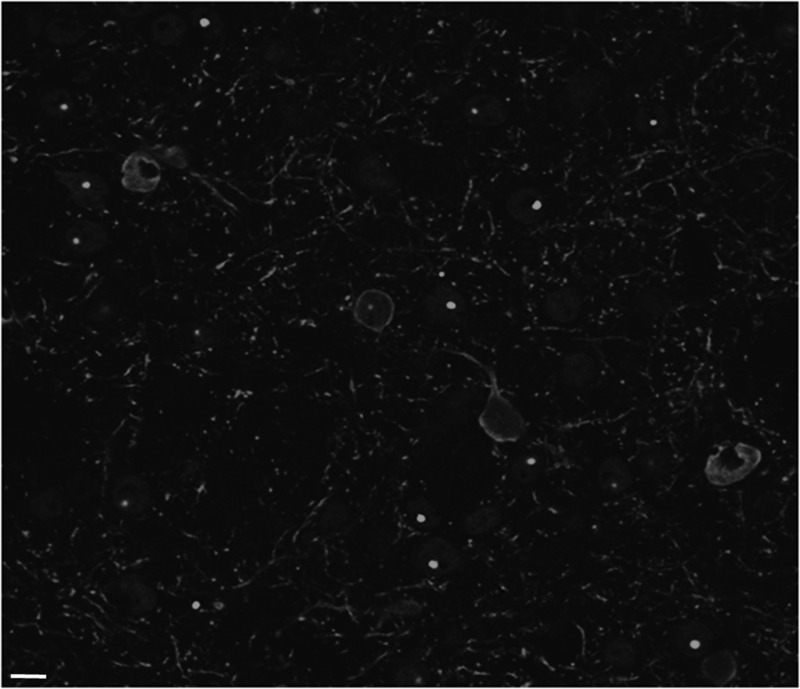
Confocal images of striatal sections confirm cytoplasmic co-localization of mHttex1 with C4 scFv-HA *in vivo*. Mouse was injected at 11 weeks, sacrificed at 20 weeks. Anti-htt EM48 (green) anti-HA (red) Bar = 20 μm. A color version of this figure is available as supplementary data at *PEDS* Online.

To confirm the cytoplasmic localization of C4 scFv in brains, fixed tissue from AAV2/1 delivery of the C4 scFv intrabody gene *in vivo* was examined using confocal microscopy. B6.HD6/1 is an inbred HD transgenic mouse strain containing Httex1 with CAG 120–125 plus ∼1 kb of upstream regulatory DNA. For this experiment, the AAV was injected directly into the striatum when the aggregates are starting to form at 11 weeks, and tissue was harvested at 20 weeks. The confocal images (Fig. 3; color supplemental Fig. S4) clearly show four cells in the field that have high expression of C4 scFv-HA, as visualized with anti-HA and a red second antibody. In all four, the center nuclear area is clear, while the red label wraps around it in the cytoplasm. The transgene in this mouse does not contain GFP, and is driven by an endogenous HTT promoter, which leads to a relatively low level of observable mHttex1 in the transgenic mouse brain compared with the cellular co-transfection experiments. Diffuse Httex1 protein is therefore not resolved in this experiment, although cells that are not expressing the scFv-HA do show bright green aggregated Httex1 protein when stained with the EM48 antibody. Equivalent confocal images from AAV2/1 delivery of V_L_12.3 into transgenic mouse brains are not available; however, the authors of the extensive *in vivo* study clearly state that the complex is localized to the nucleus *in vivo* (Southwell *et al.*, 2009).

Intrastriatal injection would not be expected to counteract the systemic phenotype of weight loss; however, disease onset could be generally accelerated if the construct has a mild toxic effect on the brain over weeks to months. Southwell *et al.* (2009) reported that while V_L_12.3 could counteract short-term effects of highly overexpressed Httex1, HDR6/2 transgenic mice injected intrastriatally with AAV2/1-V_L_12.3 showed an earlier onset of weight loss (Southwell, Fig. 9A), and acceleration of premature death (Southwell, Fig. 9E), when compared with mice that received a non-intrabody expressing AAV. We therefore assessed our data on similarly injected mice that received AAV2/1-C4 scFv produced at the same facility. We used a slightly less aggressive exon1 fragment model HDR6/1, where both the weight loss and the premature death start at a later age. It is clear from our data that intrastriatal injection of the AAV2/1-C4 scFv construct does not significantly worsen the pathogenic weight loss that characterizes late-stage disease, nor does it accelerate the start of the terminal period (Table II). This is a critical safety issue before moving forward with more global therapeutic intrabody expression.

**Table II.** Intrastriatal injection of the AAV2/1-C4 scFv construct does not accelerate the pathogenic weight loss in HDR6/1 transgenic mice

Treatment	Weight loss age of onset (mean week ± SE)	Percentage of wild-type body weight at 23 weeks	Percent dying by 25 weeks
HD sham females (*n* = 12)	19.8 ± 0.5	90.4 ± 1.2	27.8
HD-C4 females (*n* = 7)	19.3 ± 0.8	88.1 ± 1.4	14.3
HD sham males (*n* = 10)	16.2 ± 0.8	64.2 ± 0.9	50
HD-C4 males (*n* = 7)	16.1 ± 0.1	64.2 ± 1.3	0

The combined live cell imaging and injected HD transgenic mouse tissue studies clearly show that the two intrabodies elicit distinctly different subcellular distributions of target. Given multiple human and animal model studies showing transcriptional dysregulation and prominent neuronal intranuclear inclusions in HD, the strongest hypothesis to explain the differences in efficacy after intrabody gene delivery is that having the antigen–antibody complex in the nucleus is sub-optimal. It is unclear whether the downstream effect is on the formation of aggregates, or their clearance (see Southwell *et al.*, 2009; Butler *et al.*, 2012) but the overall need to consider sub-cellular localization can inform development of intrabody reagents for HD and several other degenerative diseases triggered by a breakdown in proteostasis.

At the mechanistic/structural level, the structures of the HTT N-terminal peptides complexed with V_L_12.3 and C4 scFv are now available (Schiefner *et al.*, 2011; De Genst, unpublished data). Appreciation of the extent to which the structure and post-translational modification of the highly conserved AA 1-17 domain affects the localization and possibly other protein interactions of HTT is emerging rapidly. While there are differences in the contact residues in the two intrabody–peptide complexes, these do not immediately reveal why there are such sharp differences in the cellular behavior of the two intrabodies. Although the selections were to a very similar region of the N-terminal HTT, the differences in secondary structures of the peptides that do or do not include the first three AAs of the polyQ domain might be sufficient to alter the output in a critical manner.

At the cellular screening level, as more mechanistic information (La Spada and Taylor, 2010; Zuccato *et al.*, 2010; Crook and Housman, 2011) and better cell lines (An *et al.*, 2012; Jeon *et al.*, 2012; Ward and La Spada, 2012; Kaye and Finkbeiner, 2013) are becoming available, it should be possible to design screening paradigms that utilize this information at earlier stages of the intrabody drug development process. Consideration of the need for fusion constructs can also be incorporated when comparing candidates (Butler *et al.*, 2012).

Once we are confident that we know where as well as what we need to target, antibody engineering can provide fusion protein approaches to manipulate the localization, as we have shown above. The NES experiment is largely a proof of concept, since there are issues of altered solubility, immunogenicity and kinetics of the compartmentalization in different cell types, including those where the mutant gene may be interacting with membranes. It may also be necessary to create multifunctional constructs that can target the mutant protein for degradation in addition to other localization signals (Butler and Messer, 2011; Joshi *et al.*, 2012; Messer and Joshi, 2013). However, these challenges are already being addressed in the use of antibody engineering for cancer and toxin clearance, suggesting that additional tools to tackle neurodegenerative diseases will be available for future studies as they have been to date.

## Supplementary data

Supplementary data and color figures are available at *PEDS* online.

## Funding

This work was funded by National Institutes of Health NS073415 and NS053912, the Hereditary Disease Foundation, and The High Q Foundation/Cure Huntington's Disease Initiative/CHDI. E.D.G acknowledges support from the Medical Research Council (MRC G1002272). Funding to pay the Open Access publication charges for this article was provided by RCUK.

## Supplementary Material

Supplementary Data
