# Supplementary material for: Differential nuclear localization of complexes may underlie in vivo intrabody efficacy in Huntington's disease
Source: Protein Eng Des Sel. 2014 Oct;27(10):359–63. doi: 10.1093/protein/gzu041 (PMC4191446; doi:10.1093/protein/gzu041)
Supplement: Supplementary Data [file supp_27_10_359__index.html]

Supplementary Data 

# Differential nuclear localization of complexes may underlie *in vivo* intrabody efficacy in Huntington's disease

## Supplementary Data

Supplementary Data

**Files in this Data Supplement:**

- Supplementary Data - Docx file
