# Supplementary material for: Differential nuclear localization of complexes may underlie in vivo intrabody efficacy in Huntington's disease
Source: Protein Eng Des Sel. 2014 Oct;27(10):359–63. doi: 10.1093/protein/gzu041 (PMC4191446; doi:10.1093/protein/gzu041)

**SUPPLEMENTARY FIGURE LEGENDS**

Supp. Fig. S1. Cytoplasmic GFP with C4scFv vs increased nuclear GFP with VL12.3 (Designated by arrows). ST14A cells co-transfected with Httex1-72Q-eGFP and (A) Empty vector control (B) C4scFv (C) VL12.3.Live imaging at 48H. Bar = 20μm

Supp. Fig. S2. Httex1-72Q-eGFP is predominately cytoplasmic in cells cotransfected with either empty vector control or C4 scFv compared to VL12.3 at 24H (A) and 48H time points (B). ST14A cells were co-transfected with Httex1-72Q-eGFP and RFP-NLS (to label nuclei in live cells), and either empty vector control, C4 scFv, or VL12.3. Live imaging was performed at 24h and 48h. Bar = 20μm

Supp. Fig. S3. Fusion of a nuclear export signal to VL12.3 reduces the nuclear expression of Httex1-72Q-GFP. ST14A cells co-transfected with Httex1-72Q-GFP and (A) C4 scFv (B) C4NES-scFv (C) VL12.3, and (D) VL12.3-NES Live imaging at 48H. Bar = 20μm

Supp. Fig. S4. Confocal images of striatal sections confirm cytoplasmic co-localization of mHttex1 with C4 scFv-HA *in vivo*. Mouse was injected at 11 wks, sacrificed at 20 wks. Anti-htt EM48 (green) anti-HA (red) Bar = 20μm.


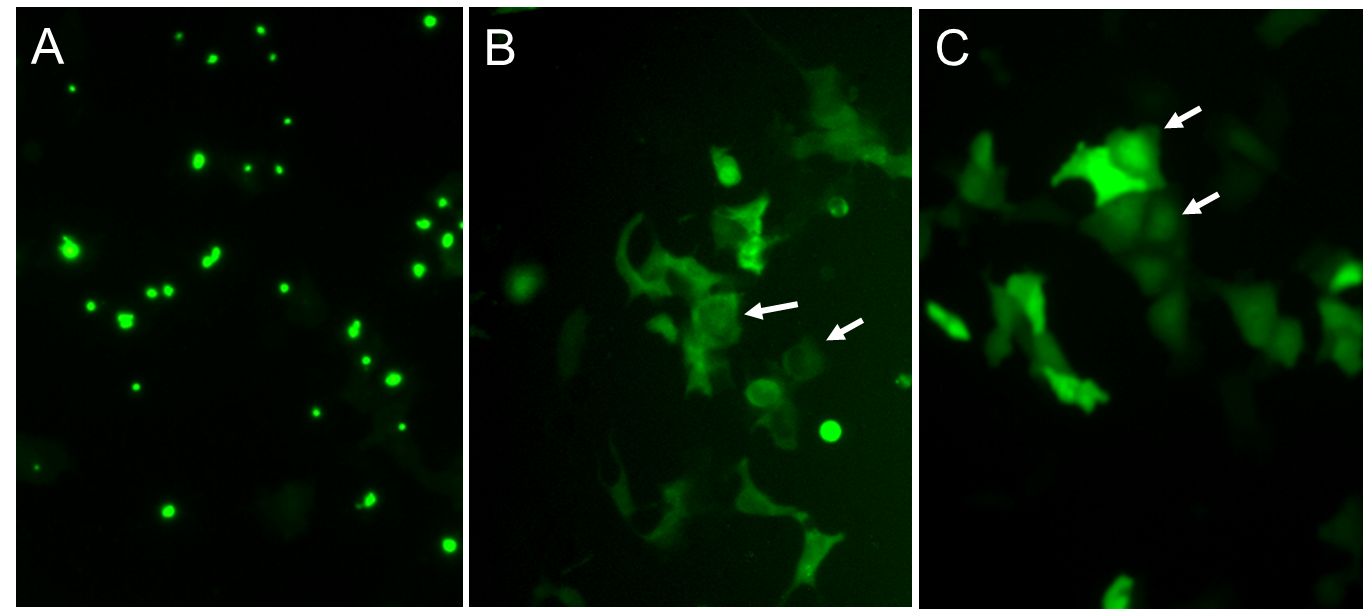


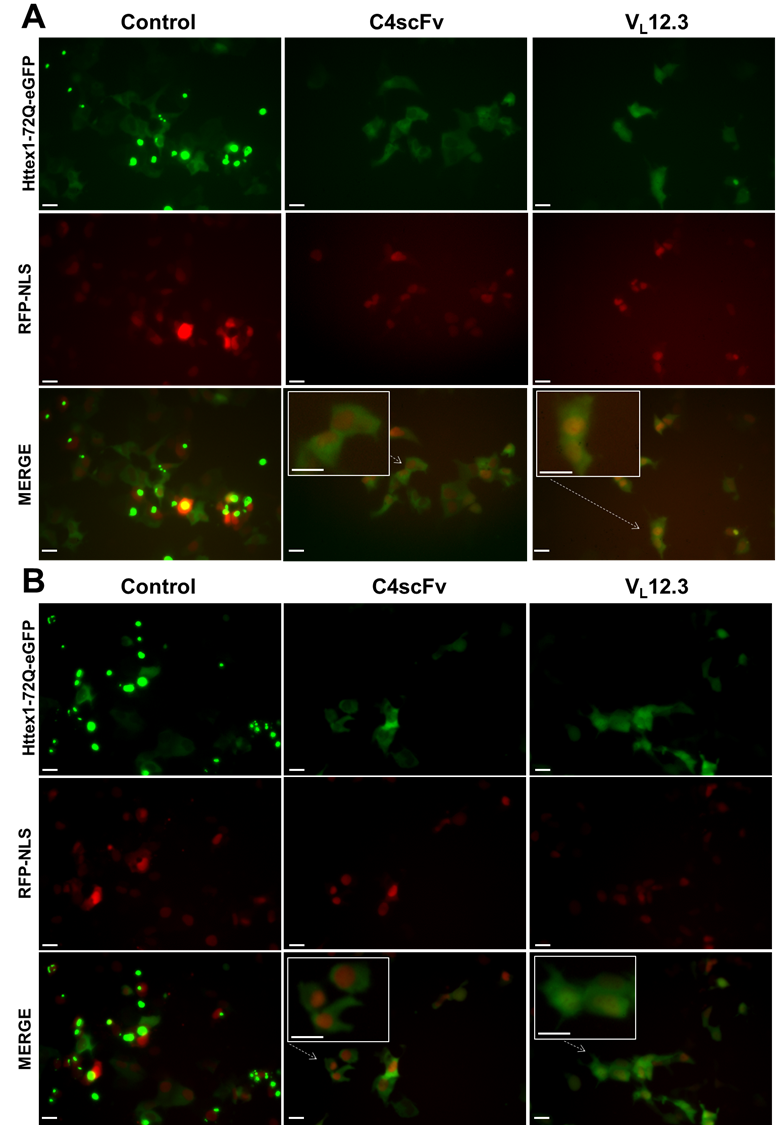


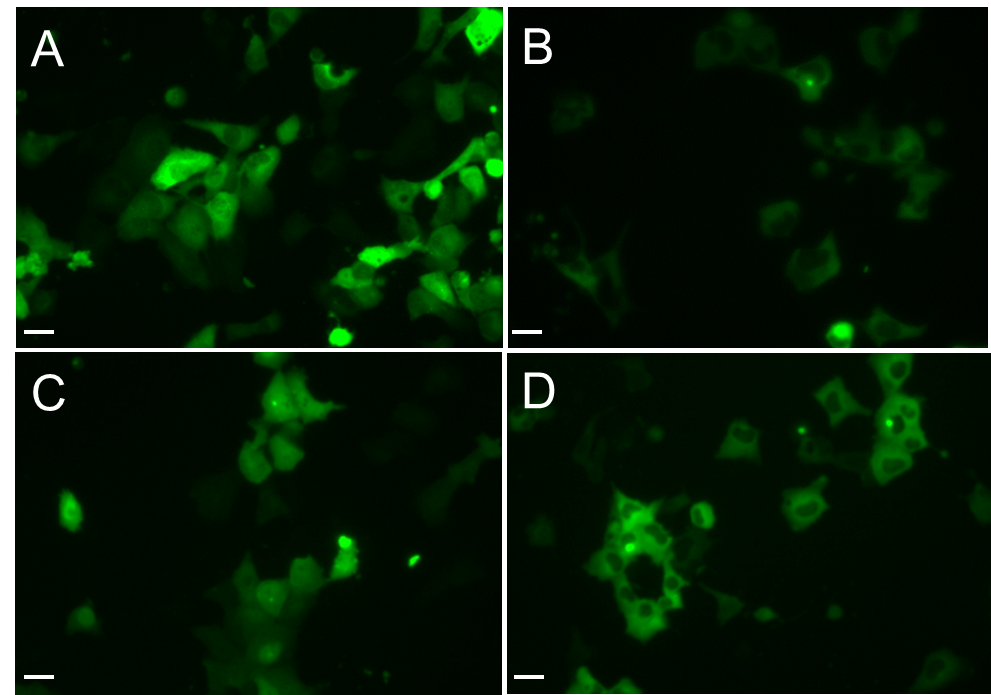


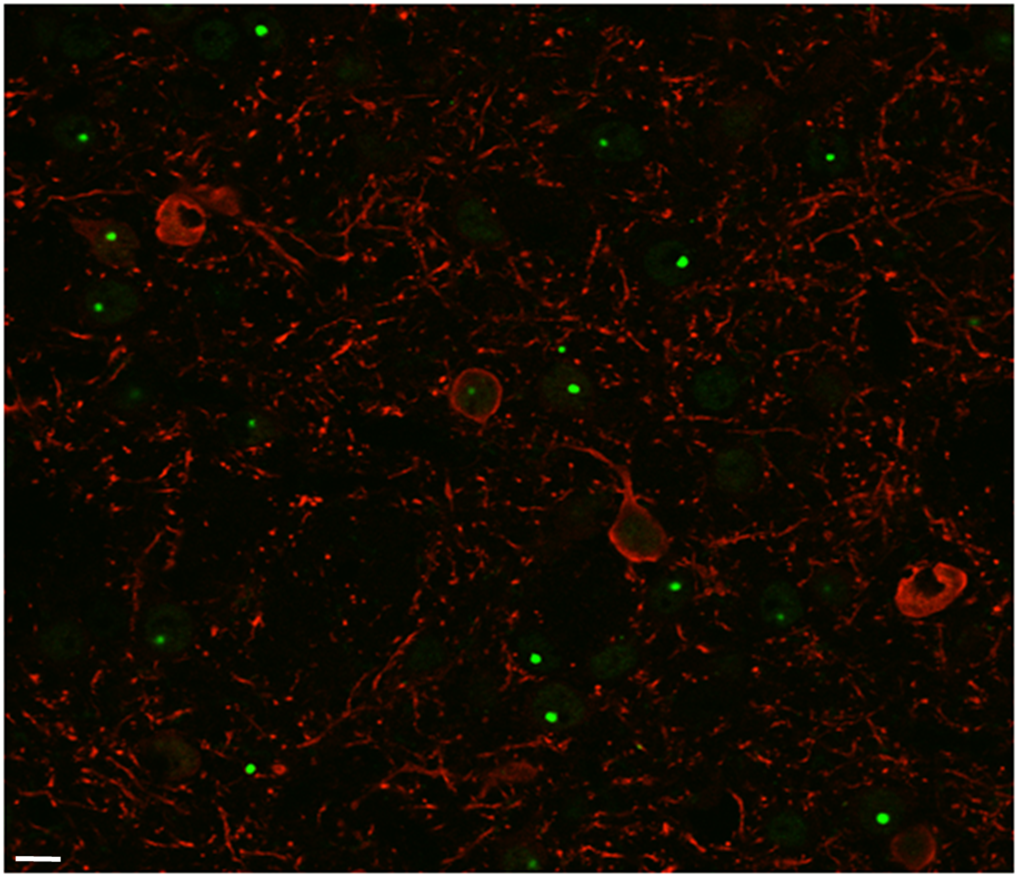

Supplement: Supplementary Data [file supp_gzu041_gzu041supp.docx]
